# Genistein Prevents Nitric Oxide Deficiency-Induced Cardiac Dysfunction and Remodeling in Rats

**DOI:** 10.3390/antiox10020237

**Published:** 2021-02-04

**Authors:** Anuson Poasakate, Putcharawipa Maneesai, Siwayu Rattanakanokchai, Sarawoot Bunbupha, Terdthai Tong-Un, Poungrat Pakdeechote

**Affiliations:** 1 Department of Physiology, Faculty of Medicine, Khon Kaen University, Khon Kaen 40002, Thailand; anuson_p@kkumail.com (A.P.); putcma@kku.ac.th (P.M.); terdthai@kku.ac.th (T.T.-U.); 2 Faculty of Veterinary Medicine, Khon Kaen University, Khon Kaen 40002, Thailand; siwara@kku.ac.th; 3 Faculty of Medicine, Mahasarakham University, Maha Sarakham 44000, Thailand; sarawoot.b@msu.ac.th; 4 Research Institute for Human High Performance and Health Promotion, Khon Kaen University, Khon Kaen 40002, Thailand

**Keywords:** genistein, cardiac function, fibrosis, MMP-2, MMP-9, collagen type I

## Abstract

Genistein is an isoflavone found in soybeans. This study evaluates the protective effects of genistein on N^ω^-nitro-L-arginine methyl ester hydrochloride (L-NAME)-induced hypertension, cardiac remodeling, and dysfunction in rats. Male Wistar rats were treated with L-NAME 40 mg/kg/day together for 5 weeks, with or without genistein at a dose of 40 or 80 mg/kg/day or lisinopril 5 mg/kg/day (*n* = 8 per group). Genistein prevented L-NAME-induced hypertension in rats. Increases in the left ventricular weight, metalloproteinase-2, metalloproteinase-9, and collagen type I intensity were observed in L-NAME rats, and these changes were attenuated in the genistein-treated group. Genistein reduced circulating angiotensin-converting enzyme activity and angiotensin II concentrations in L-NAME rats. L-NAME increased plasma and cardiac malondialdehyde and vascular superoxide generations, as well as reductions of serum and cardiac catalase activities in rats. Plasma nitrate/nitrite were protected in the genistein-treated group. Genistein prevented the L-NAME-induced overexpression of angiotensin II receptor type I (AT_1_R), nicotinamide adenine dinucleotide phosphate (NADPH) oxidase subunit 2 (gp91^phox^), and transforming growth factor beta I (TGF-β1) in hypertensive rats. In conclusion, genistein exhibited a cardioprotective effect in hypertensive rats in this study. The molecular mechanisms might be mediated by suppression of oxidative stress through the Ang II/AT_1_R/NADPH oxidase/TGF-β1 signaling pathway.

## 1. Introduction

Hypertension is a chronic disease and a major cause of organ damage and other related diseases, such as coronary artery disease, stroke, kidney disease, heart failure, etc. Chronic hypertension without optimal blood pressure (BP) control can cause cardiac hypertrophy and remodeling, which progressively develops to the impairment of cardiac contractility [1,2]. Left ventricular (LV) remodeling is mainly characterized by LV hypertrophy and fibrosis [3,4]. In an animal model of hypertension, the chronic administration of N^ω^-nitro-L-arginine methyl ester hydrochloride (L-NAME) to deplete nitric oxide (NO) production in rats has resulted in high BP, cardiac hypertrophy, and remodeling [5,6]. Bunbupha and coworkers demonstrated that L-NAME hypertensive rats have low plasma NO levels, hypertension, LV hypertrophy, and LV fibrosis [7]. Subsequently, the impairment of LV function has been demonstrated in rats that have received L-NAME, supported by a reduced maximum +LVdP/dt and minimum −LVdP/dt, ejection fraction (EF), stroke volume (SV), and fraction shortening (FS) [8,9]. There is substantial evidence to support the pathological process of cardiac remodeling associated with the renin–angiotensin system (RAS), oxidative stress, and inflammation [10,11,12,13]. Angiotensin II (Ang II) induces cardiomyocyte hypertrophy via binding to angiotensin II receptor type I (AT_1_R) and activating various intracellular signaling pathways. The activation of the RAS has been observed in NO deficiency-induced hypertension in rats [14,15]. Increased protein expression of AT_1_R in cardiac tissue associated with cardiac remodeling in hypertensive rats induced by long-term treatment with L-NAME has been confirmed [9,16].

Oxidative stress, an imbalance between reactive oxygen species (ROS) production and antioxidant defense, is believed to play a key role in hypertension and cardiovascular remodeling [17,18]. In patients with primary hypertension, endogenous antioxidant enzymatic activities are reduced [19], while plasma malondialdehyde (MDA) levels are increased [20]. Furthermore, in experimental hypertension cases, increases in plasma MDA, protein carbonyl, and vascular superoxide production and reductions in plasma superoxide dismutase (SOD) and tissue catalase (CAT) activity have been observed in L-NAME-induced hypertensive rats [21]. In fact, the link between the RAS and oxidative stress has been reported in the context of the activation of AT_1_R by Ang II, which stimulates superoxide anion (O_2_^•−^) production via NADPH oxidase subunit 2 (gp91^phox^) [22]. Rincón and coworkers showed that NO inhibition-induced hypertension is accompanied with the overexpression of the subunits gp91^phox^, p22^phox^, p47^phox^, and p67^phox^ of NADPH oxidase in renal tissue and an increase in lipid peroxidation [15]. There is evidence to show that gp91^phox^ is the most abundant isoform found in cardiac tissue and responds to stretching or Ang II to mediate ROS production and the development of LV hypertrophy [23].

It is now becoming apparent that cardiac fibrosis is an obvious feature of cardiac remodeling and hypertrophy. It is characterized by an imbalance between the synthesis and degradation of extracellular matrix (ECM) proteins. Thus, the disruption of ECM homeostasis is a crucial factor for cardiac integrity and dysfunction. Collagen types I and III are predominantly found in myocardium and represent interstitial fibrosis [24]. Matrix metalloproteinases (MMPs), a family of matrix proteases, are responsible for the degradation of ECM proteins such as elastin, fibronectins, and collagen [25]. Ang II-induced increased MMP expression and cardiac remodeling has been reported in several studies [26,27,28]. Transforming growth factor-β (TGF-β) is a fibrogenesis in myocardial cells and is activated by Ang II [29,30]. The expression of TGF-β is increased in LV tissue and is relevant to remodeling [12]. Ang II and AT_1_R are associated with oxidative stress and TGF-β1 signaling is becoming the most recognized type of signaling [29]. The accumulating data demonstrate that Ang II affects cardiac hypertrophy and fibrosis via stimulating collagen synthesis and TGF-β1 generation [31,32].

Lisinopril, an angiotensin-converting enzyme (ACE) inhibitor, is used as a positive drug in the present study. It is the most common antihypertensive drug that is used to achieve the optimal lowering of BP and treat heart failure and myocardial infarction [33,34]. Lisinopril does not contain a sulphydryl group and exhibits a long-term action duration [35]. It reduces renal vascular resistance and LV hypertrophy in essential hypertensive patients [35]. The anti-inflammatory, anti-fibrotic, and anti-angiogenic activities of lisinopril have also been reported in rats with hepatic fibrosis [36].

The soybean is one of the earliest functional foods that has been widely consumed in Asian countries [37]. A wide range of phytochemical effects in the soybean have been studied. Genistein (4′,5,7-trihydroxyisoflavone) is the main isoflavone found in the soybean. The consumption of soy milk represents a genistein intake of approximately 25.86 mg/L [38]. Genistein has exhibited several biological activities, such as the inhibition of protein tyrosine kinase (PTK) [39], along with anti-cancer, anti-inflammation, antioxidation [40,41], and anti-apoptosis activities [42]. In type 2 diabetic mice, genistein has reduced fasting blood glucose, insulin resistance, hepatic abnormalities, and inflammation associated with the adjustment of the gut microbiota composition [43]. The renoprotective effects of genistein and its mechanisms have been noted in terms of mediating the Nrf2-HO-1/NQO1 pathway in diabetic rats [44]. Genistein has prevented myocardial injury in streptozotocin-induced type 1 diabetic rats via reducing inflammation and oxidative stress [45]. Additionally, the beneficial effect of genistein on the cardiovascular system has received more attention recently since genistein has been shown to inhibit thrombotic occlusion in mouse femoral arteries and whole-blood platelet aggregation [46]. There is evidence that high soy and isoflavone intake is associated with a reduced risk of cerebral infarction and myocardial infarction in Japanese women, especially postmenopausal women [47]. This present study aims to investigate the protective effect of genistein on LV dysfunction and remodeling and the precise mechanism involved in NO inhibition-induced hypertensive rats.

## 2. Materials and Methods

### 2.1. Chemicals

Genistein (catalog number: LSM190723) was obtained from Xi’an Le Sen Bio-technology Co., Ltd. Xi’an, China. L-NAME (catalog number: N5751) and CAT enzymes (catalog number: C1345) were obtained from Sigma-Aldrich Corp. (St. Louis, MO, USA). Thiopental sodium (Reg. No.: 1C 314/47) was purchased from Anasthal^®^ (Jagsonpal, India) and lisinopril (Reg. No.: 1C 95/95) was obtained from AstraZeneca (Thailand) Ltd. Bangkok. All the other chemicals used in this experiment were obtained from standard companies and were of an analytical grade.

### 2.2. Animals

Male Wistar rats with a 180–220 g body weight (BW), 6 weeks old, were obtained from Northeast Animal Laboratory Center, Khon Kaen, Thailand. They were housed in plastic cages in a room with a regular 12 h dark–light cycle at a controlled temperature (23 ± 2 °C) at the Northeast Laboratory Animal Center, Khon Kaen University, Khon Kaen, Thailand. All animal experiments complied with the standards for the care and use of experimental animals and the present study was approved by Animal Ethics Committee of Khon Kaen University, Khon Kaen, Thailand (IACUC-KKU 75/2562).

### 2.3. Experimental Protocols

All rats acclimatized for one week. Rats were divided into six groups: Control; control treated with genistein at 80 mg/kg BW/day (control + G80); L-NAME hypertension (L-NAME); L-NAME hypertension treated with genistein at 40 mg/kg BW/day (L-NAME + G40); L-NAME hypertension treated with genistein at 80 mg/kg BW/day (L-NAME + G80); and L-NAME hypertension treated with lisinopril at 5 mg/kg BW/day (L-NAME + Lis) (*n* = 8 per group). Rats in a control group received drinking water while rats in hypertensive groups received L-NAME (40 mg/kg BW/day) in their drinking water. Genistein and lisinopril were dissolved in 10% dimethyl sulfoxide (DMSO) and propylene glycol [48]. The treatment was orally administered using a gavage feeding tube for 5 weeks. The BW and systolic blood pressure (SP) values for all rats were monitored weekly. The dose of lisinopril (5 mg/kg) was chosen based on the results from a preliminary experiment.

### 2.4. Indirect Measurement of Blood Pressure in Conscious Rats

All rats were placed in restrainers to measure SP using an indirect method. A tail-cuff plethysmography (IITC/Life Science Instrument model 229 and model 179 amplifier; Woodland Hills, CA, USA) was used to detect the SP from the tail artery of all rats.

### 2.5. Echocardiography

At the end of experiment, rats were anesthetized via an intraperitoneal injection with thiopental sodium (60 mg/kg). Their chests were shaved and cleaned, and they were placed on one side. Echocardiograms were performed using the Model LOGIQ S7 instrument (GE Healthcare, Waukesha, WI, USA). The LV structure and function were assessed using a two-dimensional short-axis view technique, and then M-mode tracings were recorded for the LV internal dimension at the end-diastole (LVIDd), end-systole (LVIDs), interventricular septum at diastole (IVSd) and systole (IVSs), LV posterior wall thickness at diastole (LVPWd) and at systole (LVPWs), end diastolic volume (EDV), end systolic volume (ESV), and SV from three consecutive cardiac cycles. LV fractional shortening (% LVFS) was calculated by the following equation: % LVFS = [(LVIDd − LVIDs)/LVIDd] × 100.

### 2.6. Hemodynamic Parameters Measurement

After cardiac function measurement, the left femoral artery was identified and cannulated by a polyethylene tube. Baseline values for the SP, diastolic blood pressure (DP), mean arterial pressure (MAP), pulse pressure (PP), and heart rate (HR) were continuously monitored for 30 min using the Acknowledge Data Acquisition software package (Biopac Systems Ins., Santa Barbara, CA, USA) with the frequency at 50 Hz.

At the end of the study, blood samples were collected from the abdominal aorta into an EDTA tube for assays of oxidative stress markers and nitric oxide metabolites (NOx) and ACE activity and Ang II levels. Both sides of the carotid arteries and heart were rapidly excised to analyze oxidative stress markers, cardiac fibrosis, and weight.

### 2.7. Immunofluorescence in Cardiac Tissue

Heart tissues were kept and washed in phosphate-buffered saline (PBS). Thereafter, the tissues were soaked in 4% paraformaldehyde for 48 h at 4 °C. After fixation, the samples were transferred to 30% sucrose in PBS at 4 °C overnight. The tissues were embedded in an embedding medium for frozen tissue specimens (catalog number: 4583, Sakura Finetek USA, Inc., Torrance, CA, USA). Blocks were cut with the Cryostat Microm HM 525 instrument (ThermoFisher Scientific, Walldorf, Germany) with a thickness of 10 µm at −25 °C and were placed on a polysine adhesive microscope slide (HDAS002). Sections of the heart were obtained following the protocols of Givvimani and colleagues [49]. The slides were processed and incubated with anti-MMP-2 (catalog number: sc-13595, Santa Cruz Biotechnology, Inc., Dallas, TX, USA) or MMP-9 (catalog number: AB19016, Merck, Germany) antibodies or anti-collagen type I (catalog number: ab34710, Abcam) at 4 °C overnight. Following overnight primary antibody application, the slides were washed with 0.1 M PBST 3 times (10 min each) and the secondary antibody (Alexa Fluor^®^ 488 (catalog number: ab150077, Abcam) or Alexa Fluor^®^ 594 (catalog number: sc-515884, Santa Cruz Biotechnology, Inc., Dallas, TX, USA)) was applied for 2 h at 37 °C. Next, the slides were washed with PBST and stained with DAPI (catalog number: D9542, Sigma-Aldrich, USA) for 10 min. In the last step, the slides were washed with PBS 3 times (5 min each), then mounted and visualized with fluorescence via a laser scanning confocal microscope (Carl Zeiss, Germany) with an appropriate filter. Confocal imaging is detected by 10X objective lens. Images were acquired with 1024 × 1024x pixels. Fluorescence intensity of DAPI, MMP-2, MMP-9, and collagen type I (average fluorescent intensity per pixel) was measured in each projection by Zeiss Zen Blue analysis wizard software.

### 2.8. Biochemical Measurements

#### 2.8.1. Assays of Serum Angiotensin-Converting Enzyme Activity

The ACE activities in the sera were measured using a fluorescence assay, following a previously described method [22] with some modifications. Briefly, 25 μL of serum was mixed with 15 mM hippuryl-L-histidyl-L-leucine (HHL) in assay buffer containing 20 mM sodium borate and 0.3 M NaCl at a pH of 8.3, with a final volume of 125 μL. The mixtures were then incubated at 37 °C for 30 min and the reaction was halted by adding 150 μL of 0.1 M NaOH. The product of the reaction was fluorogenically labeled with 10 mg/mL o-phthaldialdehyde (OPA) and read on a microplate reader at 390 nm. ACE activity was reported as mU/mL.

#### 2.8.2. Assay of Plasma Angiotensin II Concentration

The concentration of plasma Ang II was measured using an Ang II Enzyme Immunoassay (EIA) kit (RAB0010-1KT, St. Louis, MO, USA). The procedure was carried out as per the manufacturer’s instructions.

### 2.9. Measurement of Oxidative Stress Markers

#### 2.9.1. Assay of Vascular Superoxide (O_2_^•−^) Production

O_2_^•−^ production in carotid arteries was determined using lucigenin-enhanced chemiluminescence [50]. Carotid arteries were cut into 1-cm sections and then incubated with a 1-mL oxygenated Krebs-KCl solution for 30 min at 37 °C. Sample tubes with lucigenin were placed in a luminometer (Turner Biosystems, Sunnyvale, CA, USA). The production of O_2_^•−^ was shown as relative light unit counts/minute/dried weight of carotid arteries.

#### 2.9.2. Assay of Plasma and Cardiac Malondialdehyde Levels

MDA levels in plasma and cardiac tissue were measured as thiobarbituric acid reactive substances (TBARs) by a spectrophotometric method as previously described [51,52]. Briefly, a 200-mg heart apex was homogenized in 800 µL of a 0.1 M Tris–HCl buffer (pH 7.4) in an ice-cold condition. After centrifuging, the clear supernatant solution was taken to measure the MDA levels. With the measurement, 150 µL plasma or cardiac supernatant samples were reacted with the stock reagent (10% TCA, 5 mmol/L EDTA, 8% SDS and 0.5 µg/mL BHT). Before boiling, the samples were incubated for 10 min and added with 0.6% TBA. The mixture was boiled in a water bath for 30 min. After cooling, the mixture was centrifuged at 1000× *g* for 5 min. The absorbance of the supernatant was measured at 532 nm by a spectrophotometer. A standard curve was generated with concentrations from 0.3 to 10 µmol/L using 1,1,3,3-tetraethoxypropane. The values of plasma and cardiac MDA were expressed as µmol/mL plasma and nmol/mg of protein, respectively.

#### 2.9.3. Assays of Serum and Cardiac Catalase Activities

Serum CAT activity was determined by a spectrophotometric assay of H_2_O_2_, following a method from a previous publication with some modifications [53]. Briefly, 20 µL of serum was incubated in a 100-µL substrate (65 µmol/mL of H_2_O_2_ in 60 mmol/L sodium potassium phosphate buffer pH 7.4) at 37 °C for 1 min. The reaction was stopped by adding 100 µL of 32.4 mmol/L ammonium molybdate ((NH_4_)_6_Mo_7_O_24_·4H_2_O). The yellow complex was determined under a 405-nm wavelength. The value of serum CAT was expressed as kU/L.

The cardiac CAT activity was measured via the method described by Ming and coworkers with some modifications [54]. Briefly, the cardiac supernatant or the standard CAT was added into a microplate and the reaction was started by dropping 50 µL of 30% H_2_O_2_ in a 50-nM potassium phosphate buffer (pH 7.0). After that, to stop the enzymatic reaction, 25 µL of 5N H_2_SO_4_ was added and mixed thoroughly. Subsequently, 150 µL of KMnO_4_ was added and gently mixed. The activity was measured at 490 nm. The data were expressed as U/mg protein.

$$\mathrm{Serum}\;\mathrm{CAT}\;\mathrm{activity}\;(\mathrm{kU}/L)\;=\;\frac{A\left(sample\right)-A\left(blank1\right)}{A\left(blank2\right)-A\left(blank3\right)}\;\times \;271$$

### 2.10. Assay of Plasma Nitrate/Nitrite Concentrations

NOx was examined using an enzymatic conversion method followed by reaction with a Griess reagent. The absorbance of dilution was measured on an ELISA plate reader with a filter wavelength of 540 nm (Tecan GmbH., Grodig, Australia). NaNO_2_ reacted with a Griess substance produced a standard curve [55]. The value of NOx was expressed as µM.

### 2.11. Western Blot Analysis

AT_1_R, gp91^phox^, and TGF-β1 protein expressions in the heart were measured by using the Western blot method. The heart was homogenized, and the proteins were electrophoresed via a sodium dodecylsulfate polyacrylamide gel electrophoresis system. Thereafter, the proteins were electrotransferred onto a polyvinylidene difluoride membrane and blocked with 5% bovine serum albumin (BSA) in Tris-buffered saline (TBS) with 0.1% Tween-20 for 2 h at room temperature before overnight incubation at 4 °C with mouse monoclonal antibodies to AT_1_R (catalog number: sc-515884, 1:500), gp91^phox^ (catalog number: sc-74514, 1:500), and TGF-β1 (catalog number: sc-52893, 1:1000) (Santa Cruz Biotechnology, Inc., Dallas, TX, USA). After the incubation period, the membrane was washed with 3 times of TBST and then incubated with a horseradish peroxidase conjugated secondary antibody for 2 h at room temperature. The blots were developed with the Amersham^TM^ ECL^TM^ Prime solution (Amersham Biosciences Corp., Piscataway, NJ, USA) and detected using the Amersham Imager 4000 instrument (GE Healthcare Life Science, Uppsala, Sweden). The intensities of the protein bands were normalized to β-actin. The data were expressed as percentages of the values determined in the control group from the same gel.

### 2.12. Statistical Analysis

Data were expressed as mean ± the standard error of the mean (SEM). One-way analysis of variance (ANOVA) followed by Tukey testing was used to analyze the differences among groups. The data featured statistical significance when *p* < 0.05.

## 3. Results

### 3.1. Effect of Genistein on Systolic Blood Pressure in a Weekly Context

**Figure 1:**
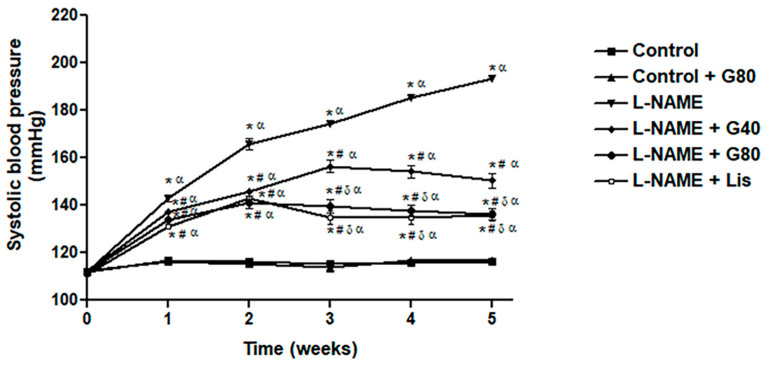
Effect of genistein or lisinopril on weekly systolic blood pressure (SP) in rats treated with N^ω^-nitro-L-arginine methyl ester hydrochloride (L-NAME). Data are expressed as mean ± SEM. * *p* < 0.05 vs. control, ^#^ *p* < 0.05 vs. L-NAME, ^δ^ *p* < 0.05 vs. G40, and ^α^ *p* < 0.05 vs. control + G80 (*n* = 8/group). Control, normotensive rats; control + G80, normotensive rats treated with genistein 80 mg/kg; L-NAME, N^ω^-nitro-L-arginine methyl ester hydrochloride; L-NAME + G40, L-NAME hypertensive rats treated with genistein 40 mg/kg; L-NAME + G80, L-NAME hypertensive rats treated with genistein 80 mg/kg; and L-NAME + Lis, L-NAME hypertensive rats treated with lisinopril 5 mg/kg.

Rats that received L-NAME showed a significant increase in SP when compared with the control or treated control rats (*p* < 0.05). Oral administration with genistein or lisinopril in L-NAME-treated rats significantly prevented the development of SP compared to untreated hypertensive rats (*p* < 0.05). Interestingly, genistein at a dose of 80 mg/kg and lisinopril significantly had a greater preventive effect comparing to genistein at a lower dose (*p* < 0.05) (Figure 1). Additionally, genistein (80 mg/kg) had no effect on SP in normal control rats.

### 3.2. Effect of Genistein on Heart Weights

To determine the effect of genistein on L-NAME-induced cardiac hypertrophy, BW, heart weight (HW,) LV weight (LVW), HW/BW, ventricular weight (VW)/BW, and LVW/BW ratios were calculated. L-NAME-treated rats had higher HW, VW, HW/BW, VW/BW, and LVW/BW ratios compared to the control rats. In addition, genistein at a dose of 80 mg/kg significantly decreased HW and VW in L-NAME rats. Genistein did not affect BW and HW in the control rats. The lisinopril-treated group significantly attenuated L-NAME-induced cardiac hypertrophy by decreasing the HW, VW, HW/BW, VW/BW, and LVW/BW ratios (Table 1).

### 3.3. Effect of Genistein on Hemodynamic Parameters

At the end of experiment, it was found that L-NAME treatment resulted in significant increases in SP, DP, MAP, PP, and HR in treated rats when compared with control rats (*p* < 0.05). Oral administration of genistein or lisinopril significantly decreased these hemodynamic parameters when compared to the untreated group (*p* < 0.05). Genistein at a higher dose showed a greater effect than a dose of 40 mg/kg in terms of the prevention of hypertension induced by L-NAME (*p* < 0.05); however, genistein had no hypotensive effects after being given to normotensive rats. All hemodynamic parameters for L-NAME rats treated with lisinopril were not different when compared with normotensive rats. Furthermore, lisinopril significantly decreased the SP, DP, MAP, and PP in hypertensive rats when compared with rats treated with genistein at a dose of 40 mg/kg (*p* < 0.05) (Table 2).

### 3.4. Effects of Genistein on Cardiac Function

**Figure 2:**
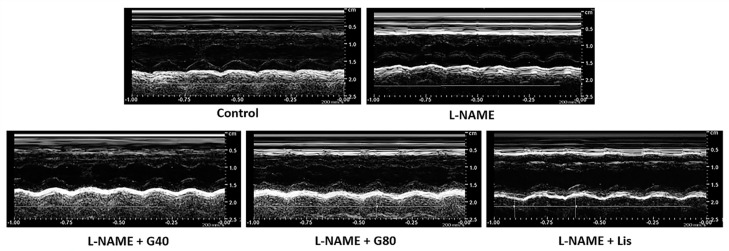
Representative M-mode echocardiographic tracings in each group, resolution 700 dpi. Control, normotensive rats; L-NAME, N^ω^-nitro-L-arginine methyl ester hydrochloride; L-NAME + G40, L-NAME hypertensive rats treated with genistein 40 mg/kg; L-NAME + G80, L-NAME hypertensive rats treated with genistein 80 mg/kg; and L-NAME + Lis, L-NAME hypertensive rats treated with lisinopril 5 mg/kg.

After administration with L-NAME for five weeks, the echocardiographic data from L-NAME rats showed significant increases in IVSd, IVSs, and LVPWd, accompanied by decreasing LVIDd, LVIDs, EDV, SV, EF, and FS values when compared with the control rats (*p* < 0.05); however, genistein or lisinopril prevented cardiac hypertrophy and dysfunction in L-NAME-induced hypertensive rats via decreasing IVSd, IVSs, and LVPWd and increasing LVIDd, LVIDs, EDV, ESV, SV, EF, and FS. (Table 3). Representative M-mode echocardiographic tracings in each group are represented in Figure 2.

### 3.5. Effect of Genistein on MMP-2, MMP-9, and Collagen Type I Protein Expression in the Heart

**Figure 3:**
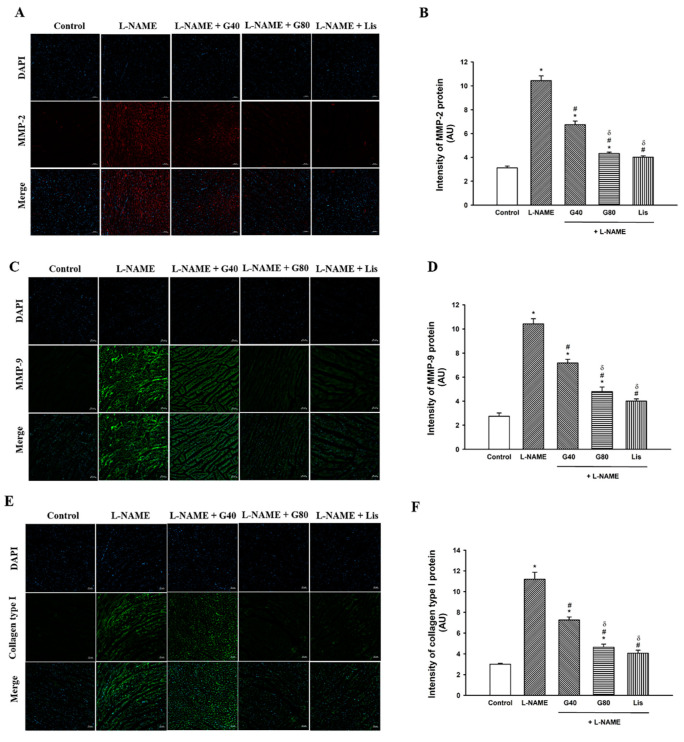
Representative images of immunofluorescence of MMP-2 (**A**), MMP-9 (**C**), and collagen type I (**E**) in the heart (magnification is ×10), resolution 800 dpi. Scale bar: 50 µm. Quantitative data for MMP-2 (**B**), MMP-9 (**D**), and collagen type I (**F**) in rats treated with L-NAME. Data are expressed as mean ± SEM. * *p* < 0.05 vs. control, ^#^ *p* < 0.05 vs. L-NAME, and ^δ^ *p* < 0.05 vs. G40 (*n* = 8/group). MMP-2, metalloproteinase 2; MMP-9, metalloproteinase 9; control, normotensive rats; L-NAME, N^ω^-nitro-L-arginine methyl ester hydrochloride; L-NAME + G40, L-NAME hypertensive rats treated with genistein 40 mg/kg; L-NAME + G80, L-NAME hypertensive rats treated with genistein 80 mg/kg; and L-NAME + Lis, L-NAME hypertensive rats treated with lisinopril 5 mg/kg.

The immunofluorescent images in Figure 3A,C,E illustrate that the fluorescent signals of the MMP-2, MMP-9, and collagen type I proteins of the LV were enhanced in the L-NAME group when compared with the control group; however, genistein or lisinopril could diminish the fluorescent signals of the MMP-2, MMP-9, and collagen type I proteins when compared with the L-NAME-induced hypertensive rats. The quantitative data for the MMP-2, MMP-9, and collagen type I proteins in Figure 3B,D,F show that the intensities of the MMP-2, MMP-9, and collagen type I proteins significantly increased in L-NAME hypertensive rats when compared with the control rats (*p* < 0.05); however, treatment with genistein or lisinopril decreased the intensities of these proteins in the myocardium in L-NAME-treated rats. The intensities of the MMP-2, MMP-9, and collagen type I proteins in hypertensive rats treated with genistein at a dose of 80 mg/kg or lisinopril were less than those when treated with genistein at a lower dosage (*p* < 0.05).

### 3.6. Effects of Genistein on Serum ACE Activity and Plasma Ang II Levels

**Figure 4:**
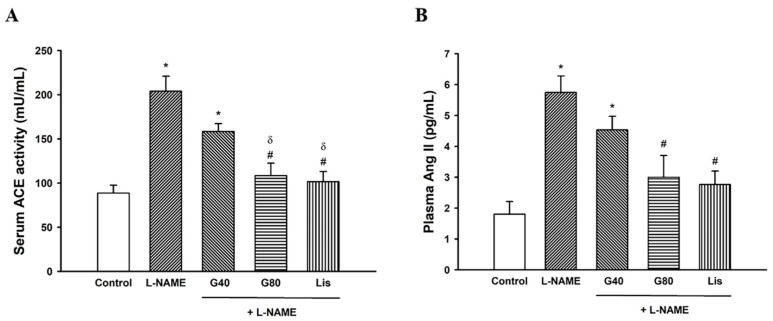
Effect of genistein or lisinopril on serum ACE activity (**A**) and plasma Ang II (**B**) in rats treated with L-NAME. Data are expressed as mean ± SEM. * *p* < 0.05 vs. control, ^#^ *p* < 0.05 vs. L-NAME, and ^δ^ *p* < 0.05 vs. G40 (*n* = 8/group). ACE, angiotensin-converting enzyme; Ang II, angiotensin II; control, normotensive rats; L-NAME, N^ω^-nitro-L-arginine methyl ester hydrochloride; L-NAME + G40, L-NAME hypertensive rats treated with genistein 40 mg/kg; L-NAME + G80, L-NAME hypertensive rats treated with genistein 80 mg/kg; and L-NAME + Lis, L-NAME hypertensive rats treated with lisinopril 5 mg/kg.

After five weeks of L-NAME administration, serum ACE activity and plasma Ang II levels were significantly increased in hypertensive rats when compared with the control rats (*p* < 0.05). Reductions of serum ACE activity and plasma Ang II levels in L-NAME-treated rats with genistein or lisinopril were observed when compared with the untreated rats (*p* < 0.05) (Figure 4). ACE activity in L-NAME rats treated with genistein (80 mg/kg) or lisinopril was significantly lower than the activity with genistein at a dose of 40 mg/kg.

### 3.7. Effects of Genistein on Oxidative Stress Markers

**Figure 5:**
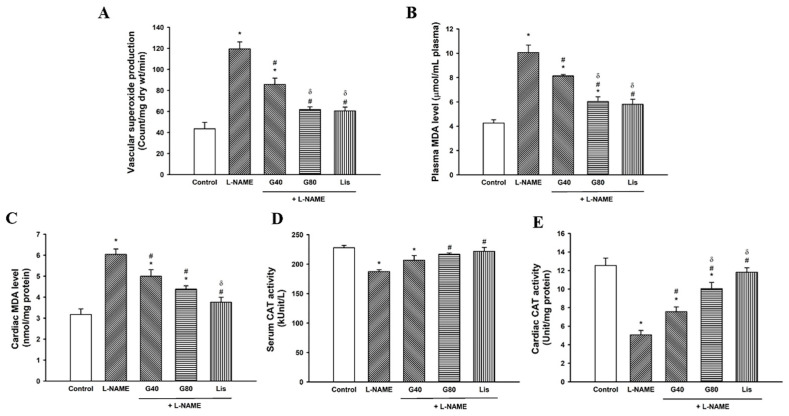
Effect of genistein or lisinopril on vascular O_2_^•−^ production (**A**), plasma MDA level (**B**), cardiac MDA level (**C**), serum CAT (**D**), and cardiac CAT activities (**E**) in rats treated with L-NAME. Data are expressed as mean ± SEM. * *p* < 0.05 vs. control, ^#^ *p* < 0.05 vs. L-NAME, and ^δ^ *p* < 0.05 vs. G40 (*n* = 8/group). O_2_^•−^, superoxide anion; MDA, malondialdehyde; CAT, catalase; control, normotensive rats; L-NAME, N^ω^-nitro-L-arginine methyl ester hydrochloride; L-NAME + G40, L-NAME hypertensive rats treated with genistein 40 mg/kg; L-NAME + G80, L-NAME hypertensive rats treated with genistein 80 mg/kg; and L-NAME + Lis, L-NAME hypertensive rats treated with lisinopril 5 mg/kg.

There were significant increases in vascular O_2_^•−^ production and plasma and cardiac MDA levels (*p* < 0.05) in L-NAME-induced hypertensive rats when compared to the control group (*p* < 0.05). These increases in O_2_^•−^ production and plasma and cardiac MDA were significantly alleviated in L-NAME rats treated with genistein or lisinopril when compared to the L-NAME group (*p* < 0.05) (Figure 5A–C). Genistein at 80 mg/kg or lisinopril produced greater antioxidant effects than genistein at 40 mg/kg in L-NAME hypertensive rats. Moreover, decreased serum and cardiac CAT activities were observed in L-NAME hypertensive rats (*p* < 0.05) when compared to the control group. Genistein (80 mg/kg) or lisinopril could restore CAT activity in hypertensive rats when comparing with the L-NAME group (*p* < 0.05) (Figure 5D,E).

### 3.8. Effect of Genistein on Plasma NOx Concentration

**Figure 6:**
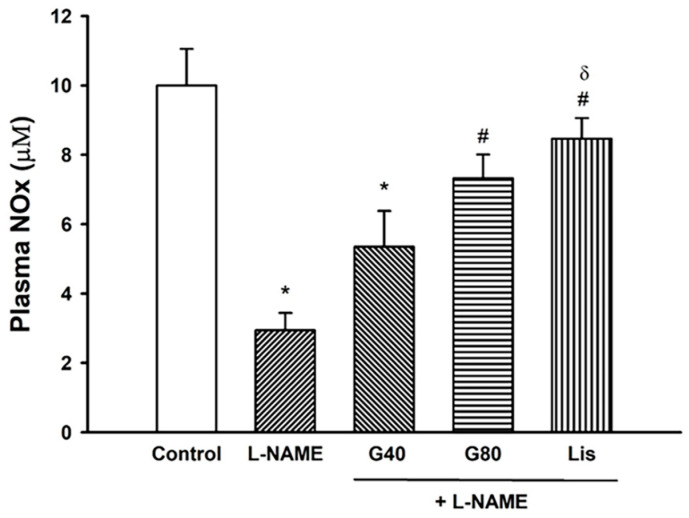
Effect of genistein or lisinopril on plasma nitric oxide metabolites (NOx) in rats treated with L-NAME. Data are expressed as mean ± SEM. * *p* < 0.05 vs. control, ^#^ *p* < 0.05 vs. L-NAME, and ^δ^ *p* < 0.05 vs. G40 (*n* = 8/group). NOx, nitric oxide metabolites; control, normotensive rats; L-NAME, N^ω^-nitro-L-arginine methyl ester hydrochloride; L-NAME + G40, L-NAME hypertensive rats treated with genistein 40 mg/kg; L-NAME + G80, L-NAME hypertensive rats treated with genistein 80 mg/kg; and L-NAME + Lis, L-NAME hypertensive rats treated with lisinopril 5 mg/kg.

Plasma NOx concentrations were reduced in the L-NAME hypertensive group and these reductions were improved and restored by treatment with genistein at a dose of 80 mg/kg and lisinopril (*p* < 0.05) (Figure 6).

### 3.9. Effect of Genistein on AT_1_R, gp91^phox^, and TGF-β1 Protein Expressions in Cardiac Tissue

**Figure 7:**
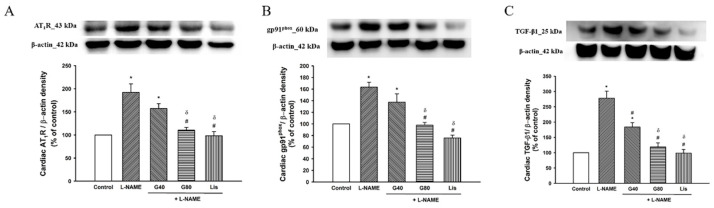
Effect of genistein or lisinopril on AT_1_R (**A**), gp91^phox^ (**B**), and TGF-β1 (**C**) in cardiac tissue in rats treated with L-NAME. Data are expressed as mean ± SEM. * *p* < 0.05 vs. control, ^#^ *p* < 0.05 vs. L-NAME, and ^δ^ *p* < 0.05 vs. G40 (*n* = 4/group). AT_1_R, angiotensin II receptor type I; gp91^phox^, nicotinamide adenine dinucleotide phosphate oxidase subunit 2; TGF-β1, transforming growth factor beta I; control, normotensive rats; L-NAME, N^ω^-nitro-L-arginine methyl ester hydrochloride; L-NAME + G40, L-NAME hypertensive rats treated with genistein 40 mg/kg; L-NAME + G80, L-NAME hypertensive rats treated with genistein 80 mg/kg; and L-NAME + Lis, L-NAME hypertensive rats treated with lisinopril 5 mg/kg.

Upregulations of AT_1_R, gp91^phox^, and TGF-β1 protein expression in cardiac tissue were observed in L-NAME-induced hypertensive rats. Treatments with genistein (80 mg/kg) or lisinopril suppressed expressions of AT_1_R, gp91^phox^, and TGF-β1 when compared with the untreated group. Genistein at a higher dose or lisinopril produced a greater effect on the suppression of the expression of AT_1_R, gp91^phox^, and TGF-β1 when comparing with genistein at a lower concentration (*p* < 0.05). Treatment with genistein at a dose of 40 mg/kg had no effect on AT_1_R and gp91^phox^ expression in L-NAME hypertensive rats (*p* < 0.05) (Figure 7).

## 4. Discussion

This study found that genistein prevents the development of hypertension and LV hypertrophy and dysfunction in rats with induced NO deficiency. LV fibrosis was supported by the high intensity of the fluorescent signals of MMP-2, MMP-9, and collagen type I protein in the L-NAME-treated rats, and these signals decreased in the genistein-treated rats. Increases in plasma Ang II and ACE activity, MDA, and vascular superoxide generation and reductions of serum and tissue CAT activities and plasma NOx concentrations in hypertensive rats were protected by genistein. Furthermore, genistein attenuated the overexpression of the intracellular protein signaling pathway (AT_1_R/gp91^phox^/TGF-β1) in LV tissue of rats induced with L-NAME. Genistein at a dose of 80 mg/kg produced a greater cardioprotective effect than a lower dose in L-NAME rats. Lisinopril was used as a positive control agent and had cardioprotective effects on L-NAME-induced hypertension and cardiac abnormalities. It also suppressed fibrogenesis, RAS activation, oxidative stress, and AT_1_R/NADPH oxidase/TGF-β1 protein expression in hypertensive rats.

It was found here that L-NAME increased BP and HR in rats. It has been established that the precise mechanisms are mediated by systemic vasoconstriction [56]. LV hypertrophy induced by L-NAME resulted in increases in the HW, HW/BW, and LVW/BW ratios in rats, and this was consistent with a previous study [9]. The echocardiography also showed LV hypertrophy, as evidenced by the reduction of IVS and LVID in L-NAME rats. LV fibrosis was supported by increasing the fluorescent signal intensity of MMP-2, MMP-9, and collagen type I in L-NAME rats. It is well known that cardiac remodeling is associated with sustained hypertension. Structural remodeling of the heart was presented by the chronic treatment with L-NAME group, resulting from the enhancement of fibrotic tissue growth [5]. MMP activation and ECM degradation are the primary responsible processes in LV remodeling [57]. The deposition of MMP-2, MMP-9, and collagen type I can imply that there is an increase in collagen degradation in the LV tissue of L-NAME rats [58]. Furthermore, abnormal remodeling of the LV collagen matrix is a major cause of an impairment in terms of cardiac contractility [59], and the present study found reductions of SV, EF, and FS in L-NAME rats. Reductions of cardiac function following chronic L-NAME treatment in rats have been reported in several studies [9,60]. It is known that the RAS plays an important role in the development of myocardial remodeling and heart failure [61]. Additionally, RAS activation has been proposed to participate in the development of hypertension in L-NAME-treated rats. The present study found that serum ACE activity, plasma Ang II, and the expression of AT_1_R in cardiac tissue were increased in L-NAME-treated rats. It might be possible that LV remodeling induced by L-NAME in the present study was mediated the overactivity of the RAS. Ang II increased the synthesis of collagen types I and III in fibroblasts, leading to thickening of the vascular wall and myocardium [61]. Furthermore, this RAS action on cardiac abnormalities in L-NAME rats was linked with oxidative stress, since the overexpression of an NADPH oxidase subunit, gp91^phox^, and TGF-β1 in cardiac tissue were observed in the present study.

Many factors such as vasoactive hormones, cytokines, and growth factors have been reported to be associated with collagen deposition in hearts [62]. AT_1_R activation enhances NADPH oxidase protein expression, ROS generation, and inflammation in instances of L-NAME-induced hypertension [15]. In this study, increases in vascular O_2_^•−^ generation and the plasma MDA concentration, as well as a decline in systemic and tissue CAT activities, were consistent with the upregulation of NADPH oxidase protein expression in cardiac tissue. It is worth mentioning that gp91^phox^ subunits are the most abundantly expressed isoforms in cardiomyocytes, which activate anion production [63]. A previous study has shown that oxidative stress stimulates MMP activity in cardiac fibroblasts [64]. Additionally, oxidative stress has been proven to be a key cellular mechanism participating in L-NAME-induced hypertension in animals. Several reports have demonstrated that L-NAME-treated rats exhibit increases in plasma MDA and protein carbonyl levels [21,65]. Oxidative stress-mediated L-NAME-induced hypertension is relevant to NO depletion, since reductions of plasma NOx have been found in L-NAME hypertensive rats [65,66]. L-NAME results in the uncoupling of eNOS, which can generate more O_2_^•−^ than NO. Additionally, O_2_^•−^ rapidly reacts with NO to produce peroxynitrite [67]. Thus, the low levels of NO in this animal model are primarily responsible for the high BP results. The limitation of this study should be noted. The expression of eNOS protein in cardiac issue should be detected to support the changes of NO concentrations in this study.

Dietary phytoestrogens that contain genistein are believed to be associated with reduced chances of hypertension in adults [68]. The cardioprotective effects of genistein in ovariectomized rats have been attributed in this regard [69]. Genistein has been shown to enhance myocyte contractility and consequently the gain of cardiac excitation–contraction coupling in guinea pig ventricular myocytes [70]. In this study, it was found that L-NAME-treated rats concomitantly receiving genistein exhibit the prevention of the development of hypertension and cardiac remodeling and the preservation of cardiac function. The antihypertensive effect of genistein found in the present study might be relevant to reducing RAS activation, since genistein reduced serum ACE activity and plasma Ang II concentrations in L-NAME rats here. This finding was supported by a previous study, where genistein reduced the expression of ACE in aortic endothelial cells, and ACE activity in sera and aortic tissues, and consequently reduced the circulating levels of Ang II in rats [71]. The inhibitory effect of genistein on ACE activity linked with a lower BP was also reported in hypertensive rats fed with fructose [72]. Subsequently, genistein had an antioxidant effect by reducing oxidative stress markers and raising endogenous antioxidant enzymes, resulting in increasing NO bioavailability. Numerous data have supported the claim that the hypotensive effect of genistein is associated with increased NO bioavailability and the expression of antioxidant enzymes [73]. The antioxidant effect of genistein was mediated by suppression of the AT_1_R/gp91^phox^ cascade in hypertensive rats here. The evidence of genistein suppressing the expression of the NADPH oxidase subunit has been observed in aortic endothelial cells from stroke-prone spontaneously hypertensive rats [74]. The possible mechanism of genistein in the prevention of cardiac remodeling induced by L-NAME in the present study was associated with the downregulation of TGF-β1 which resulted from the suppression of the Ang II/AT_1_R/gp91^phox^ cascade. TGF-β1 contributes to cell proliferation and enhances collagen synthesis to repair tissue. It is well established that Ang II induces TGF-β1 expression and leads to cardiac remodeling [75]. The results from this study could indicate the mechanism of genistein in alleviation of hypertension and cardiac alterations that might mediate AT_1_R, a surface receptor. This mechanism of action was consistent with a previous report that genistein can bind to estrogen receptors, located at plasma membrane, and activate its biological responses [76]. Genistein has been reported to exhibit a vasodilator effect through estrogen receptors in rat aorta and the main pulmonary artery [77].

Lisinopril, an ACE inhibitor, was used as a positive control substance to prevent L-NAME-induced hypertension and cardiac abnormalities in rats here. It is well known that lisinopril exhibits an antihypertensive effect via reducing circulating levels of Ang II and results in vasodilation [78]. This study has found that lisinopril has a similar effect with genistein in terms of reducing BP and cardiac alterations. Reductions in MMP-2, MMP-9, and collagen type I deposition in cardiac tissue were observed and this was consistent with the preservation of cardiac function. The relevant mechanism involved a decreasing circulating ACE activity and Ang II concentration. The downregulation of AT_1_R/gp91^phox^/TGF-β1 expression was shown in the lisinopril-treated group. Moreover, reductions in oxidative stress and increases in NO bioavailability were found in lisinopril-treated rats. Other studies have demonstrated that lisinopril has a beneficial effect on hearts by attenuating LV hypertrophy and preserving cardiac function in hypertensive patients [35].

## 5. Conclusions

In conclusion, the findings of this study indicate that genistein has cardioprotective effects in terms of L-NAME-induced cardiac remodeling and dysfunction in rats. These effects might be mediated by the inhibitory effect of genistein on the RAS and oxidative stress. The cellular mechanism is involved in the suppression of AT_1_R-NADPH oxidase/TGF-β signaling pathway in L-NAME rats.

## Figures and Tables

**Table 1:** Effect of genistein on body weight (BW) and heart weight (HW) in L-NAME-induced hypertensive rats.

Parameters	Control	Control + G80	L-NAME	L-NAME + G40	L-NAME + G80	L-NAME + Lis
BW (g)	380.13 ± 18.1	385 ± 12.26	382.88 ± 7.3	379.00 ± 10.3	372.46 ± 11.1	369.63 ± 7.6
HW (g)	1.02 ± 0.04	1.02 ± 0.01	1.14 ± 0.02 *^α^	1.04 ± 0.02	1.02 ± 0.02 ^#^	0.91 ± 0.02 ^#δ^
VW (g)	0.89 ± 0.03	0.90 ± 0.02	1.02 ± 0.03 *^α^	0.93 ± 0.03	0.92 ± 0.02 ^#^	0.83 ± 0.01 ^#δ^
HW/BW (g/g)	0.26 ± 0.00	0.27 ± 0.01	0.30 ± 0.01 *^α^	0.28 ± 0.01	0.28 ± 0.01	0.26 ± 0.01 ^#^
VW/BW (g/g)	0.24 ± 0.00	0.24 ± 0.01	0.28 ± 0.01 *^α^	0.26 ± 0.01	0.26 ± 0.01	0.24 ± 0.01 ^#^
LVW/BW (g/g)	0.18 ± 0.004	0.19 ± 0.00	0.22 ± 0.01 *^α^	0.21 ± 0.01 *	0.20 ± 0.01	0.19 ± 0.01 ^#^

Data are expressed as mean ± SEM. * *p* < 0.05 vs. control, ^#^ *p* < 0.05 vs. L-NAME, ^δ^ *p* < 0.05 vs. G40, and ^α^ *p* < 0.05 vs. control + G80 (*n* = 8/group). BW, body weight; HW, heart weight; VW, ventricular weight; HW/BW, heart weight body weight ratio; VW/BW, ventricular weight body weight ratio; LVW/BW, left ventricular weight body weight ratio; control, normotensive rats; control + G80, normotensive rats treated with genistein 80 mg/kg; L-NAME, N^ω^-nitro-L-arginine methyl ester hydrochloride; L-NAME + G40, L-NAME hypertensive rats treated with genistein 40 mg/kg; L-NAME + G80, L-NAME hypertensive rats treated with genistein 80 mg/kg; and L-NAME + Lis, L-NAME hypertensive rats treated with lisinopril 5 mg/kg.

**Table 2:** Effects of genistein on hemodynamic parameters in L-NAME-induced hypertensive rats.

Parameters	Control	Control + G80	L-NAME	L-NAME + G40	L-NAME + G80	L-NAME + Lis
SP (mmHg)	122.14 ± 2.48	121.57 ± 2.62	202.96 ± 3.96 *^α^	165.03 ± 2.17 *^#α^	149.34 ± 3.76 *^#δα^	134.77 ± 4.93 ^#δ^
DP (mmHg)	81.70 ± 3.66	77.99 ± 1.89	149.66 ± 5.26 *^α^	114.65 ± 3.26 *^#α^	102.52 ± 4.72 *^#α^	90.65 ± 5.22 ^#δ^
MAP (mmHg)	94.97 ± 2.50	92.52 ± 2.09	168.11 ± 2.91 *^α^	131.50 ± 2.23 *^#α^	117.94 ± 2.87 *^#δα^	106.36 ± 3.74 ^#δα^
PP (mmHg)	40.28 ± 0.60	43.58 ± 1.22	56.88 ± 2.73 *^α^	50.72 ±1.53 *	46.18 ± 1.60 ^#^	42.09 ± 1.95 ^#δ^
HR (beats/min)	356.05 ± 5.56	355.92 ± 8.34	442.24 ± 11.1 *^α^	383.00 ± 10.4 ^#^	372.85 ± 10.9 ^#^	374.38 ± 6.16 ^#^

Data are expressed as mean ± SEM. * *p* < 0.05 vs. control, ^#^ *p* < 0.05 vs. L-NAME, ^δ^ *p* < 0.05 vs. G40, and ^α^ *p* < 0.05 vs. control + G80 (*n* = 8/group). SP, systolic blood pressure; DP, diastolic blood pressure; MAP, mean arterial pressure; PP, pulse pressure; HR, heart rate; control, normotensive rats; control + G80, normotensive rats treated with genistein 80 mg/kg; L-NAME, N^ω^-nitro-L-arginine methyl ester hydrochloride; L-NAME + G40, L-NAME hypertensive rats treated with genistein 40 mg/kg; L-NAME + G80, L-NAME hypertensive rats treated with genistein 80 mg/kg; and L-NAME + Lis, L-NAME hypertensive rats treated with lisinopril 5 mg/kg.

**Table 3:** Effect of genistein on cardiac function in L-NAME-induced hypertensive rats.

Parameters	Control	L-NAME	L-NAME + G40	L-NAME + G80	L-NAME + Lis
IVSd (cm)	0.17 ± 0.01	0.21 ± 0.01 *	0.17 ± 0.01 ^#^	0.15 ± 0.01 ^#^	0.14 ± 0.01 ^#^
IVSs (cm)	0.25 ± 0.01	0.29 ± 0.01 *	0.25 ± 0.01 ^#^	0.24 ± 0.01 ^#^	0.23 ± 0.01 ^#^
LVIDd (cm)	0.66 ± 0.02	0.57 ± 0.01 *	0.63 ± 0.01 ^#^	0.64 ± 0.02 ^#^	0.65 ± 0.02 ^#^
LVIDs (cm)	0.38 ± 0.01	0.33 ± 0.01 *	0.35 ± 0.01	0.38 ± 0.01 ^#^	0.40 ± 0.01 ^#^
LVPWd (cm)	0.19 ± 0.00	0.24 ± 0.01 *	0.19 ± 0.00 ^#^	0.19 ± 0.01 ^#^	0.17 ± 0.00 ^#^
LVPWs (cm)	0.27 ± 0.00	0.26 ± 0.01	0.27 ± 0.00	0.26 ± 0.01	0.25 ± 0.01
EDV (mL)	0.71 ± 0.04	0.47 ± 0.02 *	0.55 ± 0.03 *	0.60 ± 0.03 ^#^	0.63 ± 0.04 ^#^
ESV (mL)	0.15 ± 0.01	0.14 ± 0.01	0.13 ± 0.01	0.15 ± 0.02	0.16 ± 0.01
SV (mL)	0.56 ± 0.03	0.33 ± 0.02 *	0.42 ± 0.03 *	0.45 ± 0.02 ^*#^	0.47 ± 0.03 ^#^
EF (%)	80.80 ± 0.45	70.15 ± 1.50 *	76.47 ± 1.94 ^#^	77.78 ± 0.88 ^#^	78.02 ± 0.65 ^#^
FS (%)	44.83 ± 0.97	36.29 ± 1.34 *	40.32 ± 1.63	42.17 ± 0.93 ^#^	43.38 ± 1.26 ^#^

Data are expressed as mean ± SEM. * *p* < 0.05 vs. control and ^#^ *p* < 0.05 vs. L-NAME (*n* = 8/group). IVSd, interventricular septum at diastole; IVSs, interventricular septum at systole; LVIDd, left ventricular internal dimension at end-diastole; LVIDs, left ventricular internal dimension at end-systole; LVPWd, left ventricular posterior wall thickness at diastole; LVPWs, left ventricular posterior wall thickness at systole; EDV, end diastolic volume; ESV, end systolic volume; SV, stroke volume; EF, ejection fraction; FS, fractional shortening; control, normotensive rats; L-NAME, N^ω^-nitro-L-arginine methyl ester hydrochloride; L-NAME + G40, L-NAME hypertensive rats treated with genistein 40 mg/kg; L-NAME + G80, L-NAME hypertensive rats treated with genistein 80 mg/kg; and L-NAME + Lis, L-NAME hypertensive rats treated with lisinopril 5 mg/kg.

## Data Availability

No new data were created or analyzed in this study.

## References

[1] Shimizu I., Minamino T. (2016). Physiological and pathological cardiac hypertrophy. J. Mol. Cell Cardiol..

[2] Ganau A., Devereux R.B., Roman M.J., de Simone G., Pickering T.G., Saba P.S., Vargiu P., Simongini I., Laragh J.H. (1992). Patterns of left ventricular hypertrophy and geometric remodeling in essential hypertension. J. Am. Coll. Cardiol..

[3] Cohn J.N., Ferrari R., Sharpe N. (2000). Cardiac remodeling--concepts and clinical implications: A consensus paper from an international forum on cardiac remodeling. Behalf of an International Forum on Cardiac Remodeling. J. Am. Coll. Cardiol..

[4] Konstam M.A., Kramer D.G., Patel A.R., Maron M.S., Udelson J.E. (2011). Left ventricular remodeling in heart failure: Current concepts in clinical significance and assessment. JACC Cardiovasc. Imaging.

[5] Pechánová O., Bernátová I., Pelouch V., Babál P. (1999). L-NAME-induced protein remodeling and fibrosis in the rat heart. Physiol. Res..

[6] Bernátová I., Pechánová O., Pelouch V., Simko F. (2000). Regression of chronic L -NAME-treatment-induced left ventricular hypertrophy: Effect of captopril. J. Mol. Cell Cardiol..

[7] Bunbupha S., Prachaney P., Kukongviriyapan U., Kukongviriyapan V., Welbat J.U., Pakdeechote P. (2015). Asiatic acid alleviates cardiovascular remodelling in rats with L-NAME-induced hypertension. Clin. Exp. Pharmacol. Physiol..

[8] Kumar S., Prahalathan P., Raja B. (2014). Vanillic acid: A potential inhibitor of cardiac and aortic wall remodeling in l-NAME induced hypertension through upregulation of endothelial nitric oxide synthase. Environ. Toxicol. Pharmacol..

[9] Wunpathe C., Maneesai P., Rattanakanokchai S., Bunbupha S., Kukongviriyapan U., Tong-Un T., Pakdeechote P. (2020). Tangeretin mitigates l-NAME-induced ventricular dysfunction and remodeling through the AT(1)R/pERK1/2/pJNK signaling pathway in rats. Food Funct..

[10] Rodríguez-Rodríguez P., Ramiro-Cortijo D., Reyes-Hernández C.G., López de Pablo A.L., González M.C., Arribas S.M. (2018). Implication of Oxidative Stress in Fetal Programming of Cardiovascular Disease. Front. Physiol..

[11] Mir S.A., Chatterjee A., Mitra A., Pathak K., Mahata S.K., Sarkar S. (2012). Inhibition of signal transducer and activator of transcription 3 (STAT3) attenuates interleukin-6 (IL-6)-induced collagen synthesis and resultant hypertrophy in rat heart. J. Biol. Chem.

[12] Maneesai P., Bunbupha S., Potue P., Berkban T., Kukongviriyapan U., Kukongviriyapan V., Prachaney P., Pakdeechote P. (2018). Hesperidin Prevents Nitric Oxide Deficiency-Induced Cardiovascular Remodeling in Rats via Suppressing TGF-β1 and MMPs Protein Expression. Nutrients.

[13] Yamazaki T., Komuro I., Yazaki Y. (1999). Role of the renin-angiotensin system in cardiac hypertrophy. Am. J. Cardiol..

[14] Zanchi A., Schaad N.C., Osterheld M.C., Grouzmann E., Nussberger J., Brunner H.R., Waeber B. (1995). Effects of chronic NO synthase inhibition in rats on renin-angiotensin system and sympathetic nervous system. Am. J. Physiol..

[15] Rincón J., Correia D., Arcaya J.L., Finol E., Fernández A., Pérez M., Yaguas K., Talavera E., Chávez M., Summer R. (2015). Role of Angiotensin II type 1 receptor on renal NAD(P)H oxidase, oxidative stress and inflammation in nitric oxide inhibition induced-hypertension. Life Sci..

[16] Sonoda K., Ohtake K., Uchida H., Ito J., Uchida M., Natsume H., Tamada H., Kobayashi J. (2017). Dietary nitrite supplementation attenuates cardiac remodeling in l-NAME-induced hypertensive rats. Nitric Oxide.

[17] Dubois-Deruy E., Peugnet V., Turkieh A., Pinet F. (2020). Oxidative Stress in Cardiovascular Diseases. Antioxidants.

[18] Rababa’h A.M., Guillory A.N., Mustafa R., Hijjawi T. (2018). Oxidative Stress and Cardiac Remodeling: An Updated Edge. Curr. Cardiol. Rev..

[19] Pedro-Botet J., Covas M.I., Martín S., Rubiés-Prat J. (2000). Decreased endogenous antioxidant enzymatic status in essential hypertension. J. Hum. Hypertens..

[20] Russo C., Olivieri O., Girelli D., Faccini G., Zenari M.L., Lombardi S., Corrocher R. (1998). Anti-oxidant status and lipid peroxidation in patients with essential hypertension. J. Hypertens..

[21] Chiangsaen P., Maneesai P., Kukongviriyapan U., Tong-Un T., Ishida W., Prachaney P., Pakdeechote P. (2020). Tangeretin ameliorates erectile and testicular dysfunction in a rat model of hypertension. Reprod. Toxicol..

[22] Maneesai P., Bunbupha S., Kukongviriyapan U., Senggunprai L., Kukongviriyapan V., Prachaney P., Pakdeechote P. (2017). Effect of asiatic acid on the Ang II-AT1R-NADPH oxidase-NF-κB pathway in renovascular hypertensive rats. Naunyn-Schmiedeberg’s Arch. Pharmacol..

[23] Byrne J.A., Grieve D.J., Bendall J.K., Li J.M., Gove C., Lambeth J.D., Cave A.C., Shah A.M. (2003). Contrasting roles of NADPH oxidase isoforms in pressure-overload versus angiotensin II-induced cardiac hypertrophy. Circ. Res..

[24] Spinale F.G. (2007). Myocardial matrix remodeling and the matrix metalloproteinases: Influence on cardiac form and function. Physiol. Rev..

[25] Deschamps A.M., Spinale F.G. (2006). Pathways of matrix metalloproteinase induction in heart failure: Bioactive molecules and transcriptional regulation. Cardiovasc. Res..

[26] Cau S.B., Guimaraes D.A., Rizzi E., Ceron C.S., Gerlach R.F., Tanus-Santos J.E. (2015). The Nuclear Factor kappaB Inhibitor Pyrrolidine Dithiocarbamate Prevents Cardiac Remodelling and Matrix Metalloproteinase-2 Up-Regulation in Renovascular Hypertension. Basic Clin. Pharmacol. Toxicol..

[27] Luchtefeld M., Grote K., Grothusen C., Bley S., Bandlow N., Selle T., Struber M., Haverich A., Bavendiek U., Drexler H. (2005). Angiotensin II induces MMP-2 in a p47phox-dependent manner. Biochem. Biophys. Res. Commun..

[28] Rizzi E., Castro M.M., Ceron C.S., Neto-Neves E.M., Prado C.M., Rossi M.A., Tanus-Santos J.E., Gerlach R.F. (2013). Tempol inhibits TGF-beta and MMPs upregulation and prevents cardiac hypertensive changes. Int. J. Cardiol..

[29] Rosenkranz S. (2004). TGF-β1 and angiotensin networking in cardiac remodeling. Cardiovasc. Res..

[30] Wenzel S., Taimor G., Piper H.M., Schlüter K.D. (2001). Redox-sensitive intermediates mediate angiotensin II-induced p38 MAP kinase activation, AP-1 binding activity, and TGF-beta expression in adult ventricular cardiomyocytes. FASEB J..

[31] Liu X., Shan X., Chen H., Li Z., Zhao P., Zhang C., Guo W., Xu M., Lu R. (2019). Stachydrine Ameliorates Cardiac Fibrosis Through Inhibition of Angiotensin II/Transformation Growth Factor β1 Fibrogenic Axis. Front. Pharmacol..

[32] Weber K.T., Díez J. (2016). Targeting the Cardiac Myofibroblast Secretome to Treat Myocardial Fibrosis in Heart Failure. Circ. Heart Fail..

[33] Chrysant S.G. (1994). Antihypertensive effectiveness of low-dose lisinopril-hydrochlorothiazide combination. A large multicenter study. Lisinopril-Hydrochlorothiazide Group. Arch. Intern. Med..

[34] Weinberger M.H. (1989). Angiotensin converting enzyme inhibitors enhance the antihypertensive efficacy of diuretics and blunt or prevent adverse metabolic effects. J. Cardiovasc. Pharmacol..

[35] Messerli F.H., Kaesser U.R. (1989). Lisinopril in the treatment of hypertension. J. Hum. Hypertens.

[36] Saber S., Goda R., El-Tanbouly G.S., Ezzat D. (2018). Lisinopril inhibits nuclear transcription factor kappa B and augments sensitivity to silymarin in experimental liver fibrosis. Int. ImmunoPharmacol..

[37] Messina M. (2010). A brief historical overview of the past two decades of soy and isoflavone research. J. Nutr..

[38] GolKhoo S., Ahmadi A.R., Hanachi P., Barantalab F., Vaziri M. (2008). Determination of daidzein and genistein in soy milk in Iran by using HPLC analysis method. Pak. J. Biol. Sci..

[39] Akiyama T., Ishida J., Nakagawa S., Ogawara H., Watanabe S., Itoh N., Shibuya M., Fukami Y. (1987). Genistein, a specific inhibitor of tyrosine-specific protein kinases. J. Biol. Chem..

[40] Ruiz-Larrea M.B., Mohan A.R., Paganga G., Miller N.J., Bolwell G.P., Rice-Evans C.A. (1997). Antioxidant activity of phytoestrogenic isoflavones. Free Radic. Res..

[41] Wei H., Wei L., Frenkel K., Bowen R., Barnes S. (1993). Inhibition of tumor promoter-induced hydrogen peroxide formation in vitro and in vivo by genistein. Nutr. Cancer.

[42] Li D.Y., Tao L., Liu H., Christopher T.A., Lopez B.L., Ma X.L. (2006). Role of ERK1/2 in the anti-apoptotic and cardioprotective effects of nitric oxide after myocardial ischemia and reperfusion. Apoptosis.

[43] Yang R., Jia Q. (2020). Genistein ameliorates inflammation and insulin resistance through mediation of gut microbiota composition in type 2 diabetic mice. Eur. J. Nutr..

[44] Jia Q., Yang R., Liu X.F., Ma S.F., Wang L. (2019). Genistein attenuates renal fibrosis in streptozotocin-induced diabetic rats. Mol. Med. Rep..

[45] Jia Q., Yang R., Liu X.F., Ma S.F. (2018). Protective Effects of Genistein on Myocardial Injury in Diabetic Rats. Sichuan Da Xue Xue Bao Yi Xue Ban.

[46] Kondo K., Suzuki Y., Ikeda Y., Umemura K. (2002). Genistein, an isoflavone included in soy, inhibits thrombotic vessel occlusion in the mouse femoral artery and in vitro platelet aggregation. Eur. J. Pharmacol..

[47] Kokubo Y., Iso H., Ishihara J., Okada K., Inoue M., Tsugane S. (2007). Association of dietary intake of soy, beans, and isoflavones with risk of cerebral and myocardial infarctions in Japanese populations: The Japan Public Health Center-based (JPHC) study cohort I. Circulation.

[48] Singh S., Dwivedi R., Chaturvedi V. (2012). Influence of Vehicles Used for Oral Dosing of Test Molecules on the Progression of Mycobacterium tuberculosis Infection in Mice. Antimicrob. Agents Chemother..

[49] Givvimani S., Narayanan N., Pushpakumar S.B., Tyagi S.C. (2014). Anti-Parstatin Promotes Angiogenesis and Ameliorates Left Ventricular Dysfunction during Pressure Overload. Int. J. Biomed. Sci..

[50] Lu F.J., Lin J.T., Wang H.P., Huang W.C. (1996). A simple, sensitive, non-stimulated photon counting system for detection of superoxide anion in whole blood. Experientia.

[51] Joukar S., Najafipour H., Haddad M., Sepehri G., Shahrokhi N., Dabiri S., Gholamhoseinian A., Hasanzadeh S. (2010). The Effect of Saffron Consumption on Biochemical and Histopathological Heart Indices of Rats with Myocardial Infarction. Cardiovasc. Toxicol..

[52] Nakmareong S., Kukongviriyapan U., Pakdeechote P., Donpunha W., Kukongviriyapan V., Kongyingyoes B., Sompamit K., Phisalaphong C. (2011). Antioxidant and vascular protective effects of curcumin and tetrahydrocurcumin in rats with L-NAME-induced hypertension. Naunyn Schmiedebergs Arch. Pharmacol..

[53] Góth L. (1991). A simple method for determination of serum catalase activity and revision of reference range. Clin. Chim. Acta..

[54] Ming M., Guanhua L., Zhanhai Y., Guang C., Xuan Z. (2009). Effect of the Lycium barbarum polysaccharides administration on blood lipid metabolism and oxidative stress of mice fed high-fat diet in vivo. Food Chem..

[55] Pakdeechote P., Bunbupha S., Kukongviriyapan U., Prachaney P., Khrisanapant W., Kukongviriyapan V. (2014). Asiatic acid alleviates hemodynamic and metabolic alterations via restoring eNOS/iNOS expression, oxidative stress, and inflammation in diet-induced metabolic syndrome rats. Nutrients.

[56] Bank N., Aynedjian H.S., Khan G.A. (1994). Mechanism of vasoconstriction induced by chronic inhibition of nitric oxide in rats. Hypertension.

[57] Takenaka H., Kihara Y., Iwanaga Y., Onozawa Y., Toyokuni S., Kita T. (2006). Angiotensin II, oxidative stress, and extracellular matrix degradation during transition to LV failure in rats with hypertension. J. Mol. Cell Cardiol..

[58] Vellaichamy E., Khurana M.L., Fink J., Pandey K.N. (2005). Involvement of the NF-kappa B/matrix metalloproteinase pathway in cardiac fibrosis of mice lacking guanylyl cyclase/natriuretic peptide receptor A. J. Biol. Chem..

[59] Weber K.T., Brilla C.G. (1991). Pathological hypertrophy and cardiac interstitium. Fibrosis and renin-angiotensin-aldosterone system. Circulation.

[60] Biwer L.A., Broderick T.L., Xu H., Carroll C., Hale T.M. (2013). Protection against L-NAME-induced reduction in cardiac output persists even after cessation of angiotensin-converting enzyme inhibitor treatment. Acta Physiol. (Oxf.).

[61] Mehta P.K., Griendling K.K. (2007). Angiotensin II cell signaling: Physiological and pathological effects in the cardiovascular system. Am. J. Physiol. Cell Physiol..

[62] Manabe I., Shindo T., Nagai R. (2002). Gene expression in fibroblasts and fibrosis: Involvement in cardiac hypertrophy. Circ. Res..

[63] Santillo M., Colantuoni A., Mondola P., Guida B., Damiano S. (2015). NOX signaling in molecular cardiovascular mechanisms involved in the blood pressure homeostasis. Front. Physiol..

[64] Siwik D.A., Pagano P.J., Colucci W.S. (2001). Oxidative stress regulates collagen synthesis and matrix metalloproteinase activity in cardiac fibroblasts. Am. J. Physiol. Cell Physiol..

[65] Bunbupha S., Pakdeechote P., Kukongviriyapan U., Prachaney P., Kukongviriyapan V. (2014). Asiatic acid reduces blood pressure by enhancing nitric oxide bioavailability with modulation of eNOS and p47phox expression in L-NAME-induced hypertensive rats. Phytother. Res..

[66] Berkban T., Boonprom P., Bunbupha S., Welbat J.U., Kukongviriyapan U., Kukongviriyapan V., Pakdeechote P., Prachaney P. (2015). Ellagic Acid Prevents L-NAME-Induced Hypertension via Restoration of eNOS and p47phox Expression in Rats. Nutrients.

[67] Xia Y., Zweier J.L. (1997). Superoxide and peroxynitrite generation from inducible nitric oxide synthase in macrophages. Proc. Natl. Acad. Sci. USA.

[68] Godos J., Bergante S., Satriano A., Pluchinotta F.R., Marranzano M. (2018). Dietary Phytoestrogen Intake is Inversely Associated with Hypertension in a Cohort of Adults Living in the Mediterranean Area. Molecules.

[69] Al-Nakkash L., Markus B., Bowden K., Batia L.M., Prozialeck W.C., Broderick T.L. (2009). Effects of acute and 2-day genistein treatment on cardiac function and ischemic tolerance in ovariectomized rats. Gend. Med..

[70] Liew R., Macleod K.T., Collins P. (2003). Novel stimulatory actions of the phytoestrogen genistein: Effects on the gain of cardiac excitation-contraction coupling. FASEB J..

[71] Xu Y.Y., Yang C., Li S.N. (2006). Effects of genistein on angiotensin-converting enzyme in rats. Life Sci..

[72] Palanisamy N., Venkataraman A.C. (2013). Beneficial effect of genistein on lowering blood pressure and kidney toxicity in fructose-fed hypertensive rats. Br. J. Nutr..

[73] Sureda A., Sanches Silva A., Sánchez-Machado D.I., López-Cervantes J., Daglia M., Nabavi S.F., Nabavi S.M. (2017). Hypotensive effects of genistein: From chemistry to medicine. Chem. Biol. Interact..

[74] Xu J.W., Ikeda K., Yamori Y. (2004). Genistein inhibits expressions of NADPH oxidase p22phox and angiotensin II type 1 receptor in aortic endothelial cells from stroke-prone spontaneously hypertensive rats. Hypertens. Res..

[75] Sun Y., Zhang J.Q., Zhang J., Ramires F.J. (1998). Angiotensin II, transforming growth factor-beta1 and repair in the infarcted heart. J. Mol. Cell Cardiol..

[76] Kuiper G.G., Lemmen J.G., Carlsson B., Corton J.C., Safe S.H., van der Saag P.T., van der Burg B., Gustafsson J.A. (1998). Interaction of estrogenic chemicals and phytoestrogens with estrogen receptor beta. Endocrinology.

[77] Mishra S.K., Abbot S.E., Choudhury Z., Cheng M., Khatab N., Maycock N.J., Zavery A., Aaronson P.I. (2000). Endothelium-dependent relaxation of rat aorta and main pulmonary artery by the phytoestrogens genistein and daidzein. Cardiovasc. Res..

[78] Thind G.S. (1990). Angiotensin converting enzyme inhibitors: Comparative structure, pharmacokinetics, and pharmacodynamics. Cardiovasc. Drugs Ther..

